# Beta-Hydroxybutyrate Mitigates Sensorimotor and Cognitive Impairments in a Photothrombosis-Induced Ischemic Stroke in Mice

**DOI:** 10.3390/ijms25115710

**Published:** 2024-05-24

**Authors:** Artem P. Gureev, Irina S. Sadovnikova, Ekaterina V. Chernyshova, Arina D. Tsvetkova, Polina I. Babenkova, Veronika V. Nesterova, Ekaterina P. Krutskikh, Daria E. Volodina, Natalia A. Samoylova, Nadezda V. Andrianova, Denis N. Silachev, Egor Y. Plotnikov

**Affiliations:** 1 Department of Genetics, Cytology and Bioengineering, Voronezh State University, 394018 Voronezh, Russia; gureev@bio.vsu.ru (A.P.G.);; 2 Laboratory of Metagenomics and Food Biotechnology, Voronezh State University of Engineering Technology, 394036 Voronezh, Russia; 3 A.N. Belozersky Institute of Physico-Chemical Biology, Lomonosov Moscow State University, 119992 Moscow, Russia

**Keywords:** stroke, ischemia, neuroprotection, mitochondria, oxidative stress

## Abstract

The consequences of stroke include cognitive deficits and sensorimotor disturbances, which are largely related to mitochondrial impairments in the brain. In this work, we have shown that the mimetic of the ketogenic diet beta-hydroxybutyrate (βHB) can improve neurological brain function in stroke. At 3 weeks after photothrombotic stroke, mice receiving βHB with drinking water before and after surgery recovered faster in terms of sensorimotor functions assessed by the string test and static rods and cognitive functions assessed by the Morris water maze. At the same time, the βHB-treated mice had lower expression of some markers of astrocyte activation and inflammation (*Gfap*, *Il-1b*, *Tnf*). We hypothesize that long-term administration of βHB promotes the activation of the nuclear factor erythroid 2-related factor 2/antioxidant response element (Nrf2/ARE) pathway, which leads to increased expression of antioxidant genes targeting mitochondria and genes involved in signaling pathways necessary for the maintenance of synaptic plasticity. βHB partially maintained mitochondrial DNA (mtDNA) integrity during the first days after photothrombosis. However, in the following three weeks, the number of mtDNA damages increased in all experimental groups, which coincided with a decrease in *Ogg1* expression, which plays an important role in mtDNA repair. Thus, we can assume that βHB is not only an important metabolite that provides additional energy to brain tissue during recovery from stroke under conditions of mitochondrial damage but also an important signaling molecule that supports neuronal plasticity and reduces neuroinflammation.

## 1. Introduction

The ketogenic diet has known effects on metabolism and nervous tissue function [1], including damage [2]. However, the effects of a ketogenic diet on the brain are primarily attributed to the appearance of ketone bodies, the best known and studied of which is beta-hydroxybutyrate (βHB) [3]. βHB is synthesized in the liver from fatty acids and then transported to other organs to meet energy needs during starvation, intense exercise, and other situations in which tissues are acutely deprived of energy, including the brain, which preferentially oxidizes carbohydrates rather than lipids [4]. During ischemia, an energy deficit is observed in the brain, but it cannot be corrected by exogenous glucose supplementation because the activation of anaerobic glycolysis leads to lactate accumulation and exacerbates ischemic damage. In contrast, exogenous administration of βHB maintains tissue ATP levels without causing lactate accumulation [5].

The metabolic effects of βHB are well known and described, but there is also some evidence that βHB is an important signaling molecule that can affect gene expression [4]. βHB inhibits class I histone deacetylase (HDAC) activity, which may contribute to increased expression of several genes [6], since histone acetylation promotes gene expression. In addition, acetylation/deacetylation directly affects the activity of many transcription factors, such as nuclear factor erythroid 2-related factor 2 (Nrf2). Nrf2 deacetylation suppresses its activity, while in the acetylated state, it can affect gene expression [7]. It is also known that βHB can inhibit glycogen synthase kinase-3β (GSK3β), which is a negative regulator of Nrf2 [8]. 

Nrf2, in turn, is a basic region leucine zipper transcription factor that binds to the antioxidant response element (ARE), thereby regulating the expression of a large battery of genes [9]. Nrf2 can activate mitochondrial biogenesis [10], protect mitochondria from oxidative damage by inducing the expression of antioxidant genes [11], activate mitophagy to remove damaged mitochondria [12], and regulate energy metabolism [13]. Ischemic tissue during stroke requires all of these processes to the highest degree. In the last decade, Nrf2 activators such as curcumin [14], dimethyl fumarate [15], resveratrol [16], sulforaphane [17], salidroside [18], chebulic acid [19], and tert-butylhydroquinone [20] have been shown to have significant therapeutic potential in brain ischemia/reperfusion.

Nrf2 not only provides mitochondrial homeostasis but also plays an important role in memory formation, as Nrf2^−/−^ mice exhibit impaired long-term potentiation due to impaired neurogenesis [21], decreased dendrite branching, decreased synaptic gene expression, and impaired cell bioenergetics in the hippocampus [22]. In turn, Nrf2 activation contributes to the improvement of cognitive performance in aging [23], lipopolysaccharide-induced neuroinflammation [24], D-galactose-induced neurotoxicity [25], high-fat diet [26], drug-induced cognitive impairment [27], streptozotocin-induced Alzheimer’s disease rat models [28], and transgenic APP/PS1 mouse (Alzheimer’s disease model) [29]. Following Nrf2 stimulation, improvements in motor coordination were observed in rotenone-induced Parkinson’s disease [30] and after spinal cord injury [31]. We hypothesize that the activation of Nrf2 target genes by βHB will contribute to the improvement of motor and cognitive functions in mice after focal ischemia, which was induced by photothrombosis (PT).

There are several models of ischemic stroke used in translational studies, with middle cerebral artery occlusion (MCAO) and PT models being the most common [32]. The main advantage of the PT model is its non-invasive nature, which produces a consistent infarct with precise localization and size. In addition, the PT model is associated with low mortality and high success rates, which is in line with modern bioethical standards. A unique feature of the PT model is the occlusion of the small cortical vessels so that the large arteries and their branches are not affected. In contrast, the intraluminal MCAO model does not take into account the thrombotic aspect that characterizes most clinical strokes in humans. Therefore, the PT model is closer to ischemic stroke in human patients. In addition, MCAO typically involves a large part of the hemisphere, resulting in a variable ischemic focus. This variability complicates the accurate sampling of brain tissue for subsequent analysis. As a result of the extensive damage to the hemisphere, severe sensorimotor and neurological dysfunction occurs, making it difficult to analyze cognitive function, which was one of the aims of this study.

The main aim of this study was to investigate the effect of βHB on sensorimotor functions and the dynamics of cognitive function recovery within 3 weeks after PT-induced stroke. We investigated the involvement of the Nrf2/ARE pathway in regulating the expression of genes involved in the maintenance of synaptic plasticity, antioxidant protection, damaged base repair systems, and the maintenance of mitochondrial DNA (mtDNA) integrity as markers of mitochondrial damage. The relationship between the expression level of inflammatory markers and cognitive and sensorimotor dysfunction in mice after focal cerebral ischemia was investigated.

## 2. Results

### 2.1. Changes in Body Weight and Feed Intake Level during the Experiment

**Figure 1:**
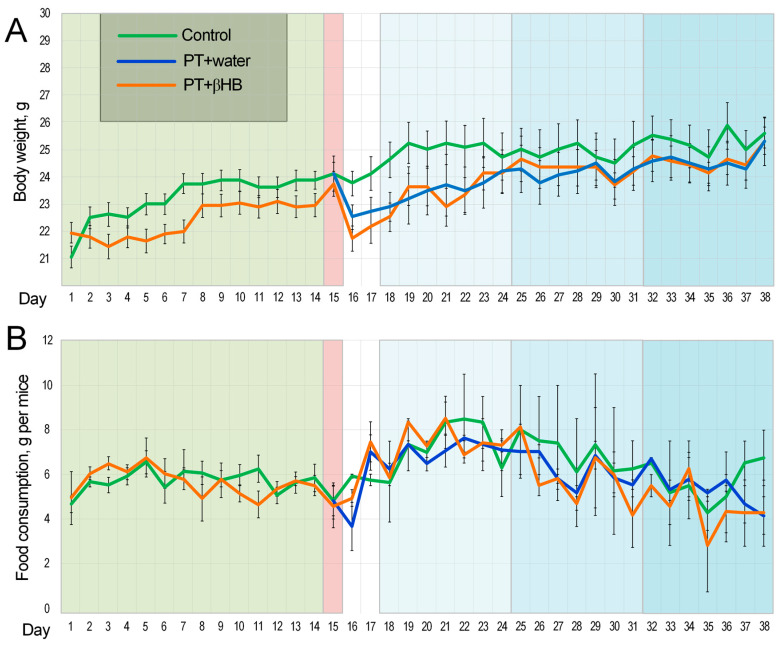
Effects of βHB and PT on body weight and food consumption: (**A**) dynamics of changes in body weight; (**B**) dynamics of changes in the level of feed intake.

The body weights of mice that consumed βHB did not differ from those of the control group mice on each day of the experiment (Figure 1A). Body weight decreased significantly in the mice on the day after PT. In the analysis of time-dependent changes in body mass on days 14–17 of the experiment, the Friedman test did not show any differences in the control group that was not subjected to PT (*p* = 0.11). Changes were noted in the groups of mice subjected to fasting and drinking water (*p* = 0.045), as well as those receiving βHB (*p* = 0.0003) (Figure 1A).

Feed intake did not differ between experimental groups but was not uniform throughout the experiment (Figure 1B). We showed time-dependent differences in feed intake in the group of mice receiving drinking water (*p* = 0.036) and βHB (*p* = 0.012). No differences in feed intake were noted in control mice (*p* = 0.19). The highest feed intake in all groups was observed during the first week of Morris water maze (MWM). In mice receiving water, feed intake was 25% higher in the first week of MWM than in the two weeks of pre-treatment (*p* < 0.001); in mice receiving βHB, it was 31% higher (*p* < 0.01). This was probably related to the increased physical activity during the MWM. Notably, feed intake normalized during the second and third weeks of the test and did not differ from the value during pre-treatment (Figure 1B). This could be related to both the adaptation of the mice to physical activity and the fact that the mice spent significantly less time searching for a platform and, consequently, undertook less physical activity in the form of swimming.

### 2.2. The Effect of βHB on Cognitive Function of Mice after Photothrombosis

**Figure 2:**
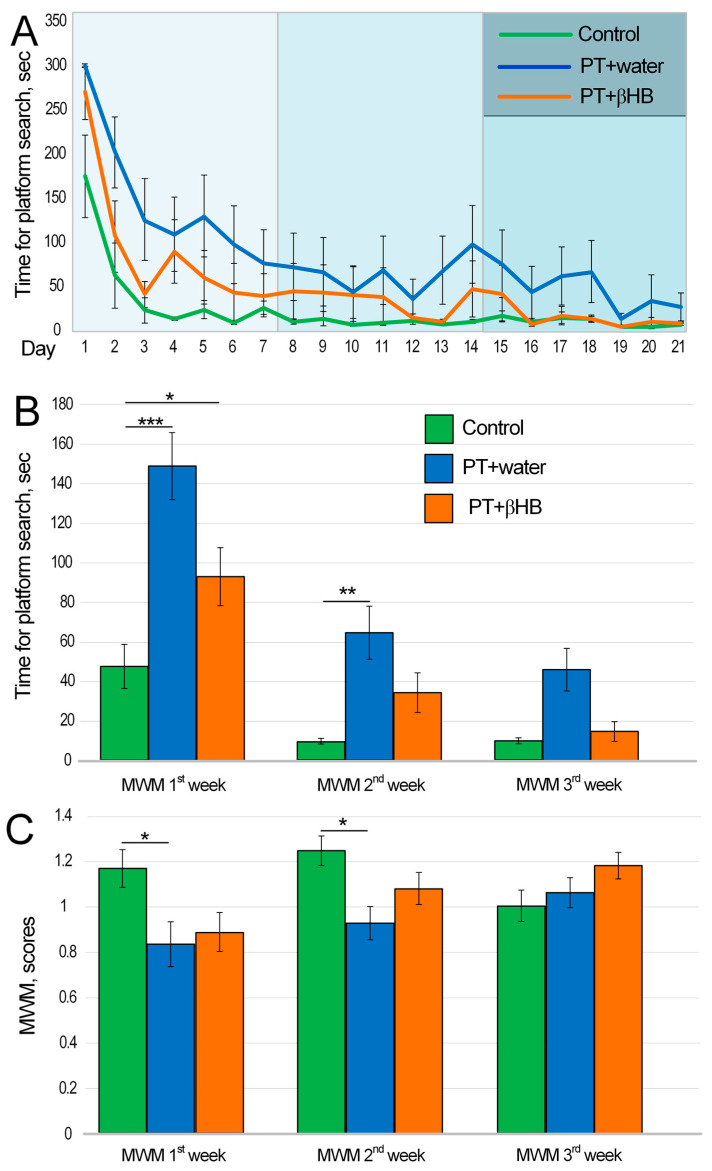
The results of MWM test conducted on the 3rd and 24th days after photothrombotic stroke in mice, which were administered either water or βHB solution. (**A**) Dynamics of time spent searching for the platform; (**B**) time spent by mice searching for the platform per week on average; (**C**) the number of points mice scored as a function of week. * *p* < 0.05, ** *p* < 0.01, and *** *p* < 0.001—differences between groups are statistically significant (Kruskal–Wallis test). The histograms for (**B**,**C**) with individual data points are provided in the Supplementary Materials (Figures S1–S6).

MWM is the best known and most commonly used test for assessing cognitive function in rodents. There are several variants of MWM protocols. The protocol used in our experiment allows for the assessment of working memory. The task for the mouse was to find the platform as quickly as possible after the first training attempt [33]. The test was performed over 3 weeks. We showed a time-dependent decrease in platform search speed day by day for the control group (*p* < 0.001), for the PT + water group (*p* < 0.001), and for the PT + βHB group (*p* < 0.001) (Figure 2A). To assess the dynamics of cognitive recovery in the mice after PT, memory scores were assessed separately for each week of the experiment. The most significant cognitive deficits were observed in the first week of testing after PT. The control group spent an average of 47.5 ± 11.2 s searching for a platform after training, whereas mice in the PT + water group spent 148.9 ± 16.8 s (*p* < 0.001) and mice in the βHB + water group spent 93 ± 14.7 s (*p* < 0.05) (Figure S1).

In the second week of testing, mice found the platform significantly faster. For example, mice from the control group found the platform in 10 ± 1.4 s. However, the mice from the PT + water group took 6.4 times longer to find the platform than those from the control group (*p* < 0.01). The mice from the PT + βHB group took 3.4 times longer, but the differences with the control group were not statistically significant (*p* = 0.11) (Figure S2). In the third week of measurements, the control mice also took 10.1 ± 1.5 s to find the platform. The time to find the platform decreased to 46 ± 10.7 s in mice from the PT + water group and 14.7 ± 4.9 s in mice from the PT + βHB group. No statistically significant differences were found between these experimental groups and the control mice. This may indicate that the cognitive deficits were attenuated by week 3 after PT, whereas the mice receiving βHB recovered their cognitive functions one week sooner (Figure 2B and Figure S3).

We also evaluated MWM results in points. Calculations took into account the difference between the speed of platform searching in the first (training) and the second (test) trials. In the first week, mice from the PT + water group scored the lowest (*p* < 0.05, compared to control mice). No differences were found between mice from the control group and those from the PT + βHB group (*p* = 0.11) (Figure S4). In the second week of measurement, the PT + water group showed a higher value, but it was still 26% lower than that of the control mice (*p* < 0.05) (Figure S5). In the third week of testing, the number of points did not differ between the different experimental groups (Figure 2C and Figure S6).

### 2.3. The Effect of βHB on Strength and Endurance Values of Mice after Photothrombosis

**Figure 3:**
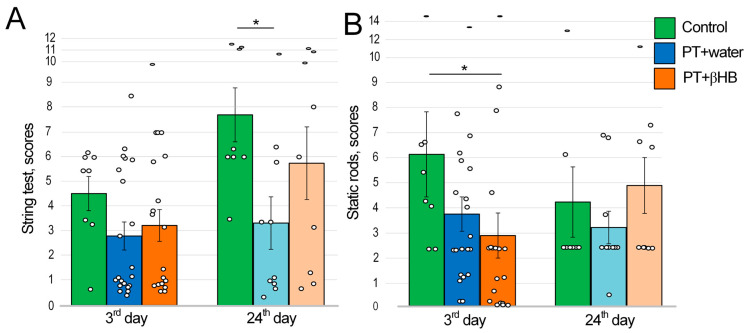
The results of the string test conducted to evaluate the strength and endurance of mice (**A**) and the static rods test conducted to assess movement coordination (**B**) on the 3rd and 24th days after photothromdotic stroke in mice, which were administered either water or βHB solution. * *p* < 0.05—differences between groups are statistically significant (Kruskal–Wallis test).

The string test allows for the assessment of motor coordination and balance, as well as muscle strength and endurance. For this, the mouse was suspended by its front paws on a string, and its behavior was evaluated with scores after 60 s of being on the string. [34]. The assessment was performed 3 days after PT. Control mice had the highest score on the sum of two attempts (4.5 ± 0.7 points), and mice from the PT + water group (2.8 ± 0.6 points) and PT + βHB group (3.3 ± 0.6 points) scored lower, but there were no statistically significant differences between groups. Twenty-four days after PT, the score in the control mice increased by 70% to 7.7 ± 1.1. In the PT + βHB group of mice, the score increased by 77% to 5.8 ± 1.5. At the same time, the PT + water group had a score of 3.4%, which was only 19% higher than that in the first measurement and significantly lower than that in the control group mice (*p* < 0.05) (Figure 3A).

### 2.4. The Effects of Photothrombosis and βHB on Movement Coordination

Static rods were used to measure coordination. A mouse’s innate response when near the end of an elevated rod is to try to reach the supported end. Scores were given according to how quickly the mouse fell from the rod or whether it reached the end of the rod [35]. The maximum score for two attempts was 6.1 ± 1.7 for the control mice. On the third day after PT, the mice showed deterioration in coordination. Mice in the PT + water group had a score of 3.8 ± 0.7 (no statistically significant difference from control); mice in the PT + βHB group had a score of 2.9 ± 0.9 (*p* < 0.05 compared to control) (Figure 3B).

Twenty-four days after PT, there were no differences in the scores. However, it is worth noting that the scores tended to decrease compared to the test at day 3 after PT. In the control group, the scores decreased from 6.1 ± 1.7 to 4.3 ± 1.4; in the PT + water group, they decreased from 3.8 ± 0.7 to 3.2 ± 0.7, whereas in the PT + βHB group, the scores increased from 2.9 ± 0.9 to 4.9 ± 1.1. These values may indicate that the differences in movement coordination were leveled 21 days after PT (Figure 3B).

### 2.5. The Effect of βHB on the Accumulation of mtDNA Damage at Different Stages after Photothrombosis

**Figure 4:**
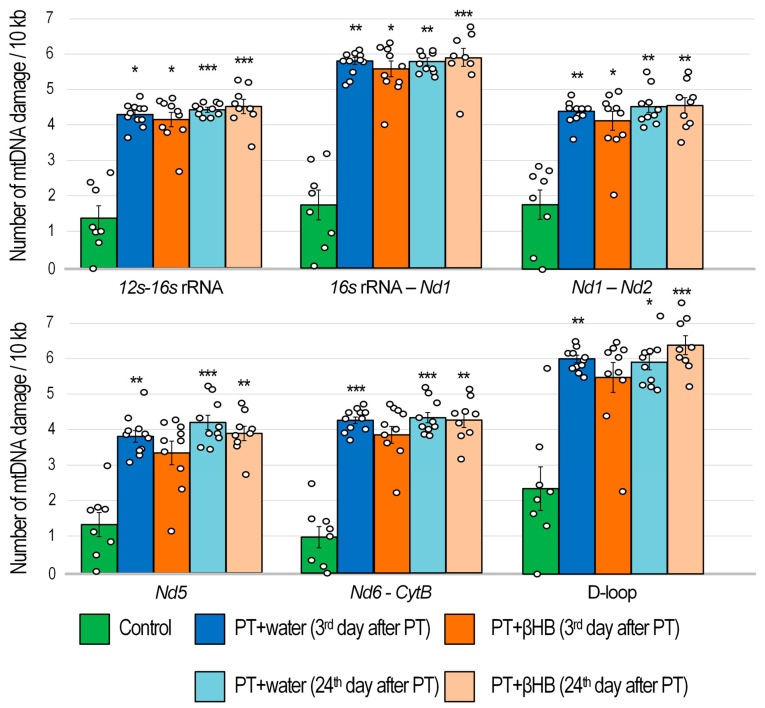
The number of damages detected via PCR in six different regions of the mtDNA isolated from the penumbra zone of mouse brain on the 3rd and 24th days after photothrombotic stroke in mice, which were administered either water or βHB solution. * *p* < 0.05 and ** *p* < 0.01, and *** *p* < 0.001—differences between groups are statistically significant (Kruskal–Wallis test).

The amount of mtDNA damage was assessed using long-range PCR. It is considered that the presence of damage reduces the amplification efficiency and the rate of accumulation of the PCR product inversely proportional to the amount of damage [36]. Photothrombosis caused a significant amount of mtDNA damage, which could be a marker for serious pathological processes in the penumbra zone. On day 3 after photothrombosis, the number of lesions in mice treated with photothrombosis was, on average, 3.3-fold higher than in mice not exposed to photothrombosis (all *p* < 0.05). In mice that received βHB solutions, on the third day after photothrombosis, the number of lesions was also 3.1-fold higher on average, depending on the mtDNA fragment. However, a statistically significant increase in the number of lesions was observed only for the mtDNA fragment encoding the *12s–16s* rRNA gene, the fragment encoding *16s* rRNA–*Nd1* (NADH dehydrogenase 1), and *Nd1–Nd2* (all *p* < 0.05). No statistically significant increase in mtDNA damage was observed in the mtDNA regions encoding the Nd5 and Nd6–*CytB* (cytochrome B) genes and in the RNA-noncoding region of the D-loop, although a similar trend was observed (*p* = 0.09, *p* = 0.06, and *p* = 0.06, respectively) (Figure 4).

Twenty-four days after photothrombosis, the number of mtDNA lesions did not decrease. On the contrary, when analyzing the total number of all DNA fragments in the PT + water group on day 24 after photothrombosis, the number of lesions was, on average, 2% higher than in the PT + water group on day 3 after photothrombosis. The differences with the control group were significant (all *p* < 0.05). The number of lesions on day 24 after photothrombosis was 11% higher in the βHB-treated mice group than on day 3 after photothrombosis. The differences with the control group were significant (all *p* < 0.01) (Figure 4).

### 2.6. The Effect of βHB on Gene Expression at Different Time Points after Photothrombosis

**Figure 5:**
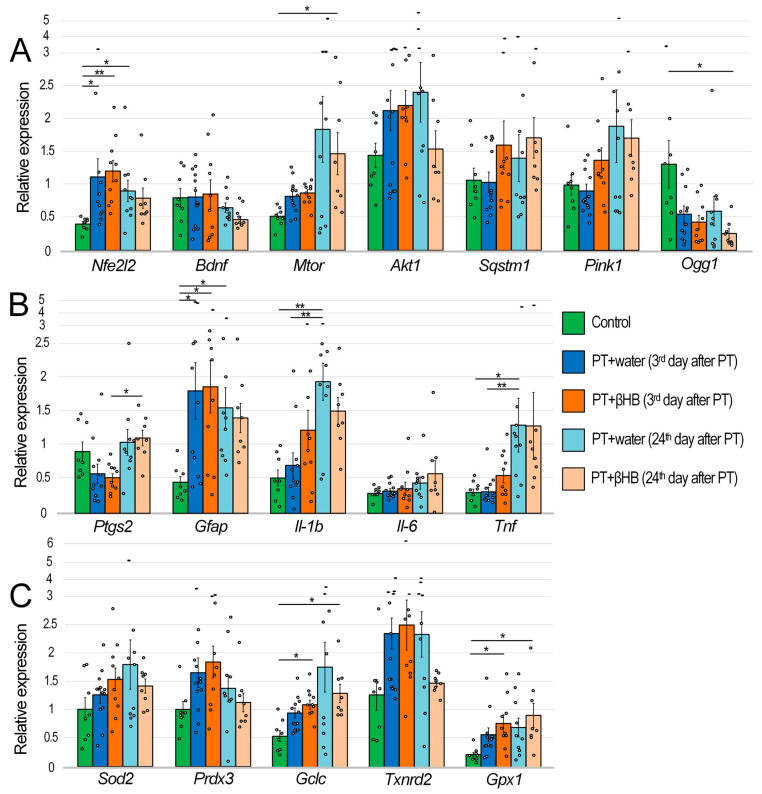
Changes in gene expression of transcription factors, regulators of mitophagy and synaptic plasticity (**A**), inflammatory markers (**B**), and antioxidant genes (**C**) in the penumbra zone of mouse brain on the 3rd and 24th days after photothrombotic stroke in mice, which were administered either water or βHB solution. * *p* < 0.05 and ** *p* < 0.01—differences between groups are statistically significant (Kruskal–Wallis test).

Photothrombosis promotes a threefold increase in the expression of the gene encoding the transcription factor Nrf2 (*Nfe2l2* gene) three days after surgery in both experimental groups (*p* < 0.05 comparing the control and PT + water groups and *p* < 0.01 comparing the control and PT + βHB groups). Twenty-four days after surgery, the increased level of *Nfe2l2* expression persisted in the PT + water group of mice (*p* < 0.05). The gene expression level for the mammalian target of rapamycin (*Mtor*) was increased threefold in the PT + βHB group 24 days after photothrombosis (*p* < 0.05). The expression level of the 8-oxoguanine DNA glycosylase gene (*Ogg1*) was reduced in photothrombosis; the differences between the control group and the PT + βHB group 24 days after photothrombosis were significant (*p* < 0.05). There were no changes in the expression level of brain-derived neurotrophic factor (*Bdnf*) and the serine/threonine kinase *Akt1*, which, like Mtor, are involved in the PI3K/Akt/mTOR signaling pathway, which plays a critical role in maintaining neuronal plasticity. Similarly, there were no changes in the expression levels of the sequestosome 1 (*Sqstm1*) and PTEN induced kinase 1 (*Pink1*) genes, which are involved in mitophagy regulation (Figure 5A).

In a number of experimental groups, there was an increase in the expression of genes that may be markers of inflammatory processes. At day 24 after surgery, the expression levels of the genes of interleukin-1β (*Il-1b*) and tumor necrosis factor (*Tnf*) were increased 3.7- (*p* < 0.01) and 4.1-fold (*p* < 0.05), respectively, compared with controls, as well as compared with mice at day 3 after photothrombosis, which also received water (both *p* < 0.01). Although similar trends were also observed in mice receiving βHB, no statistically significant increase in expression was detected compared to the control. In contrast, the expression of glial fibrillary acidic protein (*Gfap*), which is a marker of astrocyte activation during inflammation or damage, increased 4-fold after three days in the groups of mice treated with both water and βHB (both *p* < 0.05). The level of *Gfap* expression was also increased in the group of mice receiving water 24 days after surgery (*p* < 0.05), but there were no statistically significant differences between the control group and the PT + βHB group (day 3 after PT). There were no changes in the expression level of interleukin-6 (*Il-6*) gene. Increased expression of prostaglandin–endoperoxide synthase 2 (*Ptgs2*), also known as cyclooxygenase-2, could be a marker of inflammation, but no differences were found in any of the experimental groups compared with the control group, although the expression level of this gene was doubled in the PT + βHB group (24th day after PT) compared with the PT + βHB group (3rd day after PT) (*p* < 0.05) (Figure 5B).

After photothrombosis, there was a trend toward increased expression of antioxidant genes. However, statistically significant differences only in βHB-treated mice were found for the gene encoding the catalytic subunit of glutamate cysteine ligase (*Gclc*), which encodes a protein required for glutathione synthesis, and glutathione peroxidase 1 (*Gpx1*), which catalyzes the H_2_O_2_ reduction reaction (all *p* < 0.05). There were no changes in expression for the genes *Sod2*, the mitochondrial form of superoxide dismutase; *Prdx3*, the mitochondrial form of peroxiredoxin; and *Txnrd2*, the mitochondrial form of thioredoxin reductase (Figure 5C).

## 3. Discussion

The impairment of sensorimotor and cognitive parameters is an inherent consequence of stroke [37], depending on the brain region affected. In this study, we induced a focal stroke in the sensorimotor cortex region but assessed not only the neurological deficit but also the development of cognitive impairment. The string test, which measures forelimb strength and coordination [35], showed that there were no differences between control mice and mice exposed to photothrombosis on day 3 after surgery. Control mice had a 70% increase in score at the termination of experiments, whereas the PT + water group had only a 19% increase (Figure 3A), which can be explained by the increase in the physical strength and endurance of control mice through exercise involving daily swimming while performing another test, MWM. We suggest that although brain damage does not directly reduce limb strength and endurance, it abolishes the effect of training on these parameters in mice. Previously, using the rotarod test, it was shown that sensorimotor abnormalities persisted 7 days later [38] and 21 days after MCAO [39]. In focal ischemia, mild spontaneous recovery of sensorimotor parameters was observed as late as one month after photothrombosis [40]. At the same time, βHB partially neutralized the negative effect of photothrombosis, because after 3 weeks, body strength and endurance increased by 77%, which is comparable to the parameters of the control mice (Figure 5B). It is likely that βHB was an additional energetic factor that improved the muscle strength of the mice [41]. However, it is also possible that βHB improved neuroplasticity for the sensorimotor cortex of the mice, which had a positive effect on movement coordination [42].

In contrast, the rod test, which assesses movement coordination, showed the worst results in the PT + βHB group on day 3 after surgery. One of the probable reasons for this deterioration of parameters is a large loss of body weight (more than 8%) after surgery, which was typical for the mice in the PT + βHB group, while mice that received water lost only about 5% of their weight on average (Figure 1A). The weight loss with βHB administration might be directly related to its metabolic effects, since this substance simulates a ketogenic diet and its mimetics can cause a decrease in appetite and weight gain [43]. However, after 24 days, the PT + βHB group was the only group in which the score did not decrease (Figure 3B). The reason for lowering scores on the 24th day could probably be a loss of motivation, as described by Deacon et al., who developed this protocol [35]. Thus, we can say that βHB partially contributes to the acceleration of recovery of sensorimotor functions in mice after photothrombosis. Previously, ketone bodies were shown to improve sensorimotor function in a thoracolumbar mouse spinal cord injury model [44].

Despite the fact that we induced damage to the sensorimotor cortex, mice exposed to photothrombosis also exhibited pronounced cognitive deficits. This was shown using the MWM test, a classic test used to assess spatial memory and learning [35]. Impairments in memory have been repeatedly shown previously in both MCAO models [45,46,47] and photothrombosis models [48,49], including damage to the sensorimotor cortex [50]. It is likely that the focal ischemia of the sensorimotor cortex has a range of effects on other brain regions closely associated with cognitive processes [51], which may be accompanied by, for example, systemic inflammation or retrograde neuronal degeneration. In our experiment, mice treated with βHB showed more pronounced and faster recovery of cognitive functions than mice from the PT + water group. No statistically significant differences in score were found between the control group and the PT + βHB group, whereas the differences between the control group and the PT + water group persisted during the first two weeks after photothrombosis (Figure 2C). The mice from the PT + water group spent more time searching for a platform in the test trial than the control mice in the first two weeks after photothrombosis (Figure 2B). Previously, a ketogenic diet was shown to acutely improve cognitive function in patients with Down syndrome and Alzheimer’s disease [52]. 

Among the therapeutic effects of ketone bodies, researchers highlight the improvement of mitochondrial metabolism and maintenance of synaptic stability [53]. It is well known that the loss of synaptic plasticity that occurs in stroke significantly worsens the quality of life of patients [54], so its maintenance is an important component of the successful rehabilitation of patients. An important role in the maintenance of synaptic plasticity is played by the mTOR protein, which functions as part of the multiprotein complexes mTORC1 and mTORC2. These complexes are, as it were, hubs where signals from various neuronal receptors converge, including N-methyl-d-aspartate receptors (NMDAR), α-amino-3-hydroxy-5-methyl-4-isoxazolepropionic acid receptors, BDNF, and dopaminergic and metabotropic glutamate receptors (mGluRs); they are involved in signaling pathways that include phosphoinositide-dependent kinase-1 (PDK1), phosphatidylinositol 3-kinase (PI3K), Akt, etc. mTOR-containing complexes couple receptors and signaling pathways to the translation machinery to effect synaptic changes that form the basis for the formation and maintenance of memory [55]. Previous data have been reported showing that βHB stimulates *Bdnf* gene expression in the brain under conditions of glucose deficiency [56] and in the retina in diabetes [57]. Although we did not find any changes in *Bdnf* gene expression in our work, we found an increase in *Mtor* gene expression in mice that received βHB on day 24 after photothrombosis (Figure 5A), which may be the reason for the faster cognitive recovery in βHB-treated mice (Figure 2). We note that it has been previously shown that a ketogenic diet contributes to the reduction in mTOR levels in the liver and brain under normal conditions [58]. On the contrary, in injury, especially spinal cord injury, mTOR levels increased under a ketogenic diet, as did levels of Nrf2-dependent proteins [59]. Previously, we showed that the expression of *Nfe2l2* and a number of Nrf2-related genes increased in mice fed a ketogenic diet after stroke [60]. Similar results were obtained in the current work. The greatest increase in *Nfe2l2* gene expression occurred on day 3 after stroke in mice from the PT + βHB group. Although the level of *Nfe2l2* expression decreased slightly by day 24 (Figure 5A), its upregulation possibly contributed to the improvement in synaptic plasticity, including through the upregulation of *Mtor* gene expression. The direct role of Nrf2 in the regulation of *Mtor* gene expression has been shown previously [61].

**Figure 6:**
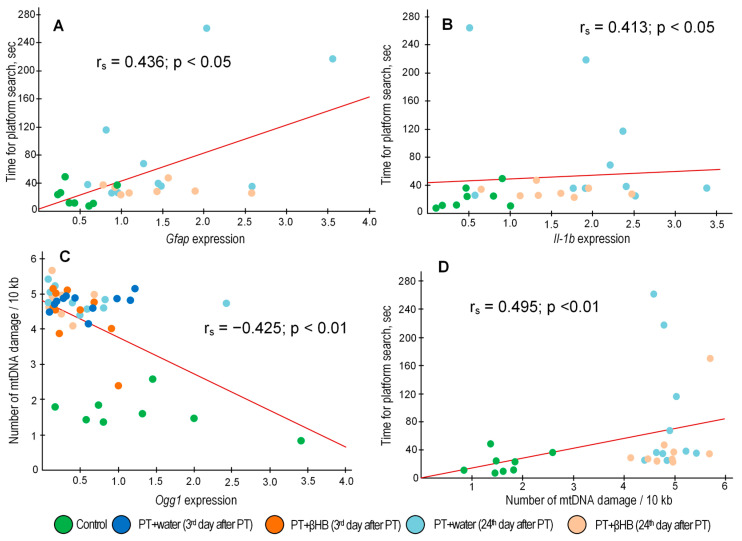
Correlation between time spent by mice searching for the platform and *Gfap* expression (**A**), between time and *Il-1b* expression (**B**), between the amount of mtDNA damage and *Ogg1* expression (**C**), and between time spent by mice searching for the platform and the amount of mtDNA damage (**D**).

Inflammation is another important factor contributing to both brain tissue damage and impaired synaptic plasticity [62]. The systemic immunity–inflammation index can be a reliable prognostic indicator of mortality and severity in stroke patients [63]. We have shown that *Gfap* expression increases significantly on day 3 after a stroke, the level of which normally increases with inflammatory responses and astrocyte activation [64], indicating the development of reactive astrogliosis. A significant positive correlation (r_s_ = 0.522; *p* = 0.002) between plasma GFAP levels and the degree of neurological deficit in patients with ischemic stroke, as measured by the National Institutional Health Stroke Scale (NIHSS) protocol, has been demonstrated previously [65]. We also performed a correlation analysis and showed a positive correlation (r_s_ = 0.436; *p* = 0.026) between the level of *Gfap* expression in the penumbra zone and the average time mice spent searching for a platform in a test trial in MWM (Figure 6A), which may indicate a relationship between photothrombosis-induced astrocyte activation and cognitive impairment in post-stroke mice. On day 24 after photothrombosis, *Gfap* expression was decreased in the group of mice receiving βHB but remained at a high level in the mice receiving water (Figure 5B). Expression levels of several other inflammatory markers, particularly *Il-1b* and *TNFα*, were significantly increased not 3 days but 24 days after photothrombosis, with statistically significant differences from controls in the PT + water group but not PT + βHB (Figure 5B), indicating a decrease in inflammation when mice were treated with βHB. Moreover, *Il-1b* gene expression levels were also positively correlated with the time of platform search in MWM (r_s_ = 0.413; *p* = 0.036) (Figure 6B). Previously, the anti-inflammatory effect of βHB has been repeatedly demonstrated in various experimental models [66,67,68], so we can assume that the positive effect of βHB on the sensorimotor and cognitive functions of mice in the post-stroke period may also be related to the anti-inflammatory effect of βHB.

The maintaining of mitochondrial stability is also critical for adequate brain function, especially for cognitive processes [69]. We have previously shown that mtDNA has accumulated a significant amount of damage in the penumbra zone 3 days after photothrombosis [60]. The amount of mtDNA damage was increased in all mtDNA fragments in the PT + water group 3 days after the surgery. However, in three of the six fragments of mtDNA examined in the brains of βHB-treated mice, there was no statistically significant increase in the number of mtDNA lesions (Figure 4), i.e., βHB slightly suppressed the accumulation of damaged mtDNA. Although we observed a gradual improvement in the cognitive and sensorimotor parameters of the mice by day 24 after PT, especially in the group of PT + βHB mice, on the contrary, the amount of mtDNA damage was slightly higher on day 24 than on day 3 after surgery in both groups (Figure 4). We can attribute this to a decrease in mtDNA repair activity, because *Ogg1* gene expression was decreased 24 days after surgery in all groups of operated mice, and βHB did not reverse this decrease (Figure 5A). OGG1 is a DNA glycosylase responsible for recognizing 8-oxoguanines and cutting DNA at a specific site to subsequently replace the damaged nucleotide via the base excision repair (BER) pathway [70]. It is considered that BER is the main pathway for mtDNA repair [71]. We found a negative correlation (r_s_ = −0.425; *p* = 0.003) between the level of *Ogg1* gene transcripts and the average number of mtDNA damages (Figure 6C), indirectly confirming the key role of OGG1 in mtDNA repair. Meanwhile, mtDNA integrity also appears to play an important role in maintaining synaptic plasticity, because we found a positive correlation (r_s_ = 0.495; *p* = 0.009) between the amount of mtDNA damage and the time that mice spent searching for a platform in the MWM (Figure 6D).

**Figure 7:**
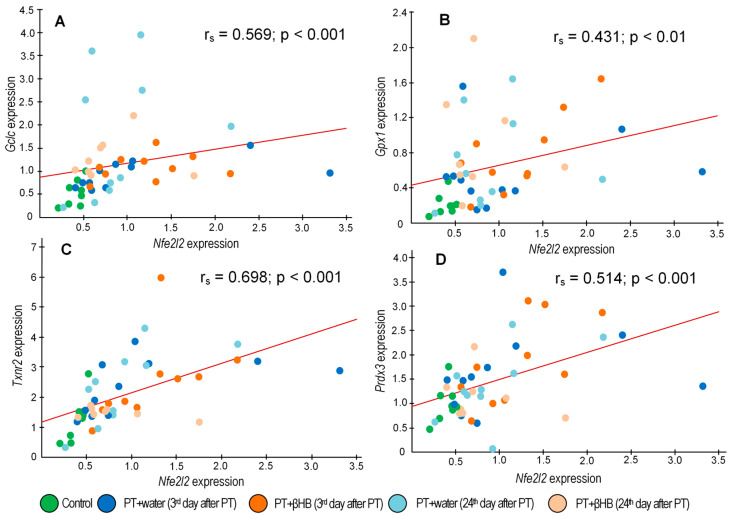
Correlation between *Nfe2l2* and *Gclc* expression levels (**A**), *Nfe2l2* and *Gpx1* expression levels (**B**), *Nfe2l2* and *Txnrd2* expression levels (**C**), and *Nfe2l2* and *Prdx3* expression levels (**D**).

Another factor affecting neuronal plasticity is the antioxidant status in the brain [72]. Reactive oxygen species (ROS) produced during photothrombic stroke appeared to cause a slight compensatory increase in most antioxidant genes studied. At the same time, a statistically significant increase in the expression of the *Gclc* gene was observed in mice treated with βHB (Figure 5C). The product of this gene encodes the first and rate-limiting step of GSH synthesis [73], the level of which plays a crucial role in the prognosis and dynamics of brain recovery after stroke [74]. Similarly, βHB contributed to the increased expression of *Gpx1* (Figure 5A), whose product scavenges H_2_O_2_ and uses GSH for this purpose [75]. We hypothesize that the activation of the Nrf2 pathway was the cause of the change in the expression of antioxidant genes. In addition, we found a positive correlation between the expression levels of *Nfe2l2* and *Gclc* (r_s_ = 0.569; *p* = 0.00003) and between the expression levels of *Nfe2l2* and *Gpx1* (r_s_ = 0.431; *p* = 0.002) (Figure 7A,B).

It is worth noting that a strong positive correlation was found between the expression levels of *Nfe2l2* and *Txnrd2* (r*s* = 0.698; *p* = 0.00000005) and between *Nfe2l2* and *Prdx3* (r_s_ = 0.514; *p* = 0.0002) (Figure 7C,D). These genes are important because they encode proteins that target mitochondria and provide antioxidant protection specifically in the mitochondrial matrix [76,77]. Nrf2 has previously been shown to control the expression of the genes *Txnrd1* and *Prdx1/2* [78], which are the cytoplasmic forms of thioredoxin reductase and peroxiredoxin, respectively. For genes encoding the mitochondrial forms of these antioxidant enzymes, Nrf2 regulation has been demonstrated for the first time. The prediction of transcription factor binding sites with FIMO revealed the presence of three probable AREs in the mouse *Prdx3* gene. However, it should be kept in mind that promoter analysis and expression correlation analysis only suggest and do not prove that Nrf2 regulates the expression of *Txnrd2* and *Prdx3*. Further studies are needed, for example, using chromatin immunoprecipitation (ChIP) or electrophoretic mobility shift assay analysis.

We should mention some limitations of this study. The cognitive impairments detected using the MWM could be related not only to memory impairments but also to motor dysfunctions, which could have contributed to the increased time taken to find the platform. Similarly, βHB may have effects not only in the brain but also in other organs, such as the muscles, which may also contribute to the interpretation of our test results and requires further investigation.

## 4. Materials and Methods

### 4.1. Animals

Male C57BL/6 mice (Stolbovaya, Moscow region, Russia) were maintained under standard conditions at a temperature of 25 °C, a 12 h light/12 h dark cycle, and a relative humidity of at least 40%. Water and a standard laboratory chow (MEST, Moscow, Russia) were available *ad libitum*. The keeping, experimental study, and sacrifice of animals were approved by the Institutional Committee for the Care and Use of Animals of Voronezh State University (Department of Animal Care and Use, Protocol on Biomedical Research 42-03, 8 October 2020).

### 4.2. Experimental Design

Male C57BL/6 mice were divided into two groups. The first group received drinking water (n = 31), and the second group received βHB at a concentration of 2 g/kg/day (n = 20). This pre-treatment was performed for 14 days. On day 15, all mice in the βHB group and 23 mice from the control group were subjected to PT (PT + water group). Eight mice were not subjected to PT and remained as the control group. On the day after the PT procedure, the mortalities of 2 mice from the PT + water group and 1 mouse from the PT + βHB group were detected.

**Figure 8:**
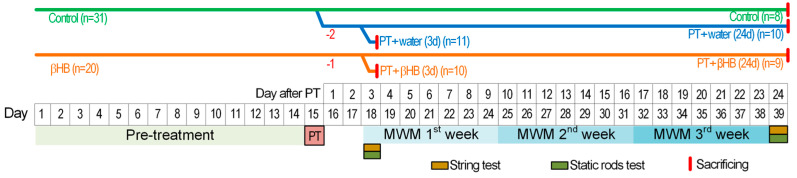
Time-line of the experiment.

Some of the mice were sacrificed 3 days after PT. These mice were used to form the PT + water (3d) (n = 11) and PT + βHB (3d) (n = 10) groups and subjected to a string and static rod tests before being killed. The other group of mice was put to death on day 24 after the PT procedure. From these mice, the groups PT + water (24d) (n = 10) and PT + βHB (24d) (n = 9) were formed. On the same day, a control group of mice that did not undergo the PT procedure was sacrificed (n = 8). Mice in these three groups were subjected to open field, string, and static rod tests on days 3 and 24 after PT. From day 3 to day 23 after PT, mice were subjected to MWM to assess working memory (Figure 8).

### 4.3. Induction of Photothrombosis

Focal ischemic stroke was modeled in the sensorimotor cortexes of mice using photochemically induced thrombosis of cerebral cortical vessels according to the Watson method [79]. Under isoflurane anesthesia (1% in air) using the SomnoSuite^®^ system (Kent Scientific Corporation, Torrington, CT, USA), the photosensitive dye Rose Bengal (3%, 40 mg/kg intravenously; Sigma-Aldrich, St. Louis, MO, USA) was injected into the jugular vein. The mouse head was then fixed in a stereotaxic frame, the skull was exposed via an incision along the midline, and the periosteum was removed. After 5 min of dye injections, the right cerebral hemisphere in the sensorimotor prefrontal cortex was continuously irradiated with a green laser at λ = 550 nm (~100 mW, 3 mm beam diameter) for 10 min. After suturing the skin, the mice were housed in a cage under an infrared heating lamp until they awoke from anesthesia. Body temperature was maintained at 37 ± 0.5 °C throughout the experiment using a rectal temperature sensor with feedback regulation. For further experiments, the penumbra area was used.

### 4.4. Morris Water Maze (MWM)

**Figure 9:**
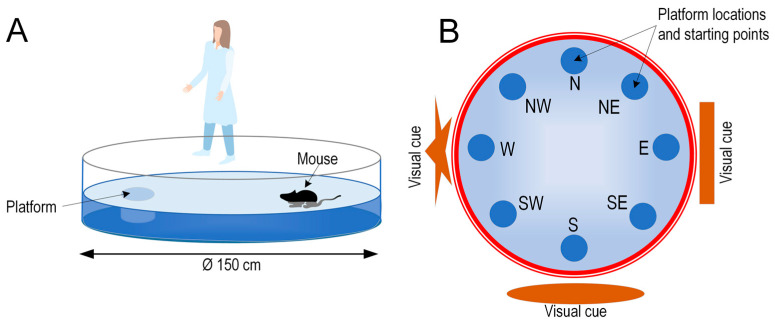
Schematic of the water tank for the Morris water maze (side view) (**A**) and top view (**B**).

The apparatus consisted of a rubber water tank with a diameter of 150 cm and a height of 33 cm. The platform, which had a diameter of 15 cm, was at the same height as the water. The pool was at least half filled with water at room temperature. The water was tinted with titanium dioxide food coloring so that the mice could not see the bottom. The entire pool was conditionally divided into four directions: N (north), S (south), E (east), and W (west). This division helped us to determine the starting point for the mice and the location of the platform. We used a protocol that assessed spatial working memory [33]. In this procedure, also known as matching-to-sample, the platform is repositioned each day, and the animal receives two trials per day. On each day, the first five-minute trial represents a sample trial. The animal must learn the new location of the platform during the first trial. The second five-minute trial is the test trial. The second trial begins after a 15 s pause. If the animal remembers the trial, it will swim a shorter path to the target on the second trial. Since the platform is moved daily, learning the position of the platform from the previous day cannot be transferred to the next day. Therefore, the test measures only temporary or working memory. The organizational scheme of the MWM is shown in Figure 9. The order in which the mice were launched and the location of the platform are shown in Table 1. 

We used two parameters to estimate spatial working memory. The first parameter is the total time (in seconds) that it took the mouse to find the platform on the second trial. If the mouse did not find the platform on the second trial, it was assigned a value of 300 s. The second parameter is the number of points (from 0 to 2 points). In total, 0 points were assigned if the mouse did not find the platform on either of its two attempts. Moreover, 0.01 to 0.99 points were assigned if the mouse found the platform more slowly on the second attempt than on the first. The calculation was based on the following formula:

Scores = (1 − Trial 2 (s)/Trial 1 (s)) + 1

Thus, for example, if the mouse found a platform twice as fast on the second attempt than on the first attempt, it would receive 1.5 points. Two points was an ideal but unrealistic situation in which the mouse did not take 0 s to find the platform. 

### 4.5. String Test

This test was used to determine the animal’s strength, endurance, and coordination of movements. The experiment was performed according to the method described in [34], albeit with modifications. The endurance of the animals was tested on a 40 cm long string attached vertically at a height of 50 cm. Each mouse was suspended alternately with its front paws in the middle of the string. The condition of the animal was scored in points after 60 s according to the following scheme: 1 point for the mouse holding on to the string with two paws, 2 points if the mouse held on to the string with two paws and moved along the string, 3 points for two front paws and one or two hind paws, and 4 points for four paws and a tail on the string.

If the mouse fell, it received 0 to 0.9 points, depending on how long (in seconds) the mouse held onto the string before falling. The calculation was made according to the following formula:

Scores = (time to fall)/60

When the mouse reached the end of the string and reached the poles supporting the string, the mouse received between 5 and 5.9 points, depending on how long (in s) it took to reach the top of the string. The calculation was made according to the following formula:

Scores = 5 + (1 − (time to escape)/60)

Each mouse was given two attempts with an interval of 30 min. Scores from both trials were summed up.

### 4.6. Static Rods

The static rod test was used to measure coordination in mice [35]. For the test, 5 rods with diameters of 35 mm, 28 mm, 22 mm, 15 mm, and 9 mm and an identical length of 60 cm were used. Each rod was clamped to the edge of the table. A “target line” was drawn 10 cm from the table. The rods were fixed 60 cm above the floor. First, the mouse was placed with its nose away from the table on the outermost edge of the 35 mm rod. The time that it took the mouse to fall or reach the finish line was scored. If the mouse fell, the trial was over. If the mouse did not make it to the finish line within 120 s, the trial was over. If the mouse made it to the finish line, it was transferred to a smaller diameter stick in the same manner. If the mouse fell, the number of points was calculated according to the formula:

Scores = 1.2 − (1.2 − (time to falling)/100)

If the mouse stayed on the platform for 120 s, it received 1.2 points. When the mouse reached the finish line, the number of points was calculated according to the following formula:

Scores = 1.2 + (time to escape)/100

The scores that the mouse received for each of the rods were summed up. Each mouse had two attempts 30 min apart. The scores from both attempts were summed up.

### 4.7. Nucleic Acids Extraction

DNA was extracted from the cortexes (half of the penumbra zone, around 0.01 cm^3^) of mice using the PROBA-GS kit (DNA Technology, Moscow, Russia) according to the protocol. RNA from mouse cortexes (second half of the penumbra zone) was isolated using the ExtractRNA reagent (Evrogen, Moscow, Russia), and further fractionation was performed using chloroform. Qualitative 8 evaluation of nucleic acid samples was performed via electrophoresis in 2% agarose gel in 1x TAE buffer.

### 4.8. Estimation of the Amount for mtDNA Damage

The amount of mtDNA damage was evaluated via long-range PCR using the Encyclo Plus PCR kit (Evrogen, Moscow, Russia) on a CFX96™ Real-Time System thermocycler (Bio-Rad, Hercules, CA, USA) [36]. Damages were assessed in six mtDNA fragments that corresponded to the *12s-16s* rRNA genes (PCR fragment position ChrM: 298–2036), *16s* rRNA-*Nd1* (ChrM position: 2078–3403), *Nd1-Nd2* (ChrM position: 3318–4992), *Nd5* (ChrM position: 11,775–13,717), *Nd6* to *CytB* (ChrM position: 13,650–15,381), and D-loop (ChrM position: 15,361–15,369). The primer sequences are presented in Table 2. To normalize the mtDNA copy number, a short mtDNA fragment was amplified (position ChrM: 2078–2174). The PCR conditions were as follows: total denaturation in 5 min at 95 °C, then 35 cycles of 10 s at 95 °C, 30 s at 61 °C, and 270 s at 72 °C.

The number of damages were calculated according to the following formula:

Damage=1 − (2(^−(Δlong − Δshort)^) × 10,000)/(fragment length)

### 4.9. Measuring of Gene Expression

To obtain cDNA on RNA matrix, the RIVERTA-L kit (AmlpiSens, Moscow, Russia) was used. The reaction was performed according to the protocol on a BIS M111-02-48 amplifier (NovosibBioPribor, Novosibirsk, Russia). Quantitative PCR analysis was performed on a CFX96^TM^ Real-Time System thermocycler (Bio-Rad, Hercules, CA, USA) using a qPCRmix-HS SYBR kit (Evrogen, MoscowRussia). PCR conditions were as follows: total denaturation in 5 min at 95 °C, then 45 cycles of 10 s at 95 °C, 30 s at 59 °C, and 30 s at 72 °C. The normalized expression level was calculated using the formula 2^(−ΔΔCq)^. The glyceraldehyde 3-phosphate dehydrogenase gene (*Gapdh*) was used as a reference. The primer sequences of the genes studied are listed in Table 3.

### 4.10. Statistical Analysis

Power analysis was performed using the STATISTICA 12 software package (StatSoft, Inc., Tulsa, OK, USA), with a significance level of 0.05. The calculated power was at least 0.8. The results are presented as means ± S.E.M. The normality of the data distribution was assessed using the Shapiro–Wilk test. Since the data distribution differed from normal, we used non-parametric statistical methods. The statistical significance of differences between groups was assessed via the Kruskal–Wallis test. Time-dependent differences between parameters in different experimental groups were evaluated using the Friedman Test/ANOVA. Correlation analysis was performed using Spearman’s rank correlation coefficient (r_s_). Statistical significance was considered to be *p* < 0.05. Transcription factor binding sites were searched with the FIMO tool (version 5.5.2). Scans were performed along the forward and reverse DNA strands; a significance threshold of *p* < 0.0001 was established. Positional weight matrices for human and mouse Nrf2 from the JASPAR open database were used.

## 5. Conclusions

Our findings suggest that the ketone body βHB may have neuroprotective effects in focal ischemic stroke, potentially improving cognitive and sensorimotor outcomes in mice within three weeks after stroke. It is well known that ketone bodies can restore tissue ATP levels and normalize mitochondrial metabolism. These beneficial effects could be mediated by both the metabolic effects and modulation of Nrf2/ARE signaling pathways associated with suppression of inflammation, increased antioxidant defenses, and enhanced mitochondrial tolerance for damage. Thus, ketone body therapy may be a promising approach for the rehabilitation of neurological and mental functions in patients after ischemic stroke.

## Figures and Tables

**Table 1:** Sequence of start and goal positions for spatial working learning and memory.

Day	Start	Goal	Day	Start	Goal	Day	Start	Goal
1st Week			2nd Week			3rd Week		
1	N	SE	8	E	SE	15	N	SE
2	E	NE	9	W	NW	16	S	SW
3	S	SW	10	S	SE	17	N	NE
4	W	SE	11	E	SW	18	S	NW
5	S	NE	12	N	SW	19	E	NW
6	N	NW	13	E	NW	20	W	SW
7	W	NE	14	W	NE	21	N	SE

**Table 2:** Primer sequences for estimating the amount of mtDNA damage.

Fragment	Forward Primer 5′–3′	Reverse Primer 5′–3′
*12s–16s rRNA*	TAAATTTCGTGCCAGCCACC	ATGCTACCTTTGCACGGTCA
*16s rRNA-Nd1*	CGAGGGTCCAACTGTCTCTTA	CCGGCTGCGTATTCTACGTT
*Nd1-Nd2*	CTAGCAGAAACAAACCGGGC	TTAGGGCTTTGAAGGCTCGC
*Nd5*	TCATTCTTCTACTATCCCCAATCC	TGGTTTGGGAGATTGGTTGATG
*Nd6–CytB*	TCATTCTTCTACTATCCCCAATCC	GGTGGGGAGTAGCTCCTTCTT
D-loop	AAGAAGGAGCTACTCCCCACC	GTTGACACGTTTTACGCCGA
Short fragment	CGAGGGTCCAACTGTCTCTTA	AGCTCCATAGGGTCTTCTCGT

**Table 3:** Primer sequences for gene expression estimation.

Gene	Forward Primer 5′–3′	Reverse Primer 5′–3′
*Gapdh*	GGCTCCCTAGGCCCCTCCTG	TCCCAACTCGGCCCCCAACA
*Akt1*	TGATCAAGATGACAGCATGGAGTG	GATGATCCATGCGGGGCTT
*Bdnf*	AAGGACGCGGACTTGTACAC	CGCTAATACTGTCACACACGC
*Gclc*	GGGGTGACGAGGTGGAGTA	GTTGGGGTTTGTCCTCTCCC
*Gfap*	CAACGTTAAGCTAGCCCTGGACAT	CTCACCATCCCGCATCTCCACAGT
*Gpx1*	AGTCCACCGTGTATGCCTTCT	GAGACGCGACATTCTCAATGA
*Il-1b*	TTGCGGACCCCAAAAGATG	AGAAGGTGCTCATGTCCTCA
*Il-6*	CGGAGAGGAGACTTCACAGAG	CATTTCCACGATTTCCCAGA
*Mtor*	AGATAAGCTCACTGGTCGGG	GTGGTTTTCCAGGCCTCAGT
*Nfe2l2*	CTCTCTGAACTCCTGGACGG	GGGTCTCCGTAAATGGAAG
*Ogg1*	GAGACGACAGCCAGGTGTGAG	CCGTTCCACCATGCCAGTA
*Sqstm1*	GCCAGAGGAACAGATGGAGT	TCCGATTCTGGCATCTGTAG
*Pink1*	GAGCAGACTCCCAGTTCTCG	GTCCCACTCCACAAGGATGT
*Prdx3*	GTGGTTTGGGCCACATGAAC	TGGCTTGATCGTAGGGGACT
*Ptsg2*	AGTCCGGGTACAGTCACACTT	TTCCAATCCATGTCAAAACCGT
*Sod2*	CAGACCTGCCTTACGACTATGG	CTCGGTGGCGTTGAGATTGTT
*Tnf*	TATGGCTCAGGGTCCAACTC	GGAAAGCCCATTTGAGTCCT
*Txnrd2*	GATCCGGTGGCCTAGCTTG	TCGGGGAGAAGGTTCCACAT

## Data Availability

The data generated and analyzed during the current study are available from the corresponding author on reasonable request.

## Supplementary Materials

The supporting information can be downloaded via this link: https://www.mdpi.com/article/10.3390/ijms25115710/s1.
