# Supplementary material for: Beta-Hydroxybutyrate Mitigates Sensorimotor and Cognitive Impairments in a Photothrombosis-Induced Ischemic Stroke in Mice
Source: Int J Mol Sci. 2024 May 24;25(11):5710. doi: 10.3390/ijms25115710 (PMC11172083; doi:10.3390/ijms25115710)
Supplement: Supplementary file 1 [file ijms-25-05710-s001.zip › ijms-2970807-supplementary.pdf]

Beta-hydroxybutyrate Mitigates Sensorimotor and Cognitive Impairments in a Photothrombosis-induced Ischemic Stroke in Mice

Artem P. Gureev, Irina S. Sadovnikova, Ekaterina V. Chernyshova, Arina D. Tsvetkova, Polina I. Babenkova, Veronika V. Nesterova, Ekaterina P. Krutskikh, Daria E. Volodina, Natalia A. Samoylova, Nadezda V. Andrianova, Denis N. Silachev, Egor Y. Plotnikov

Supplementary Figures

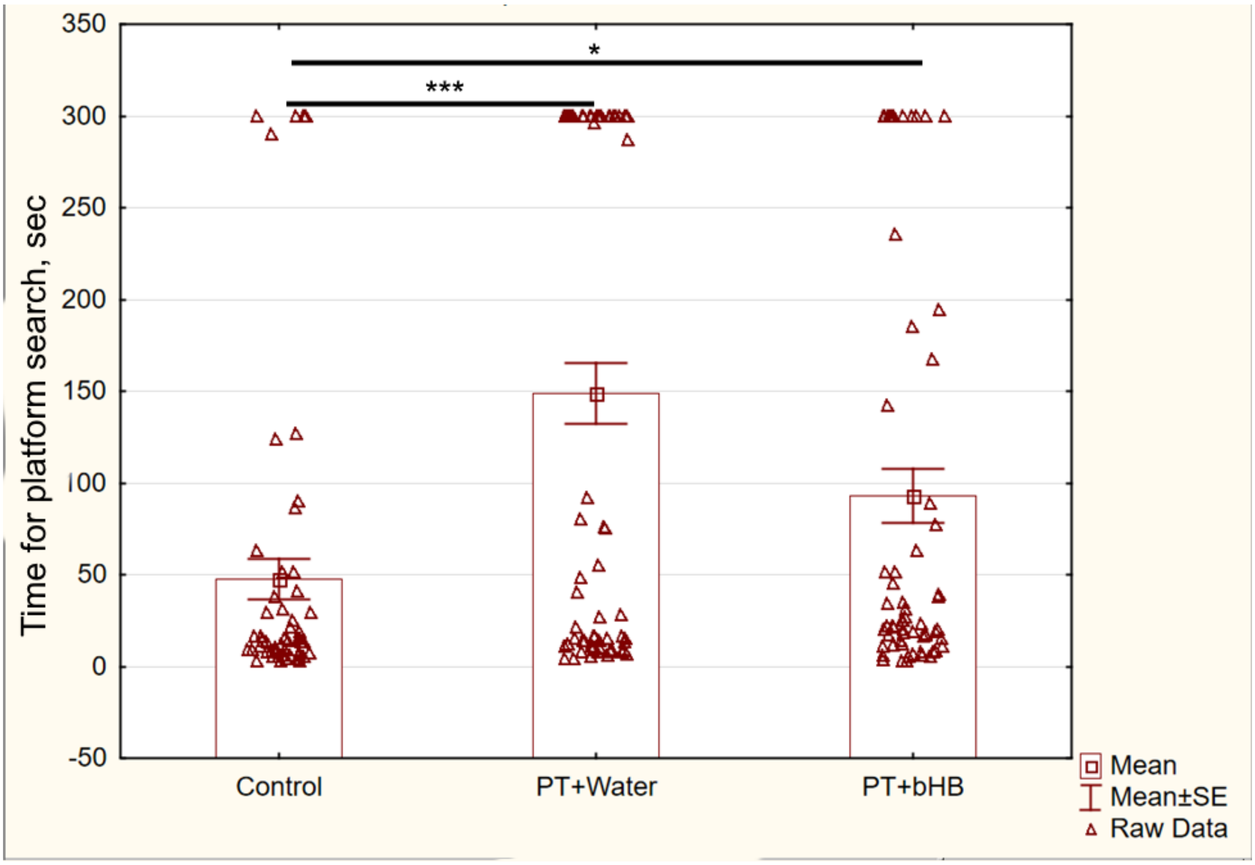

Supplementary Figure S1. Time spent by mice searching for the platform in the MWM test at the 1st week after photothrombotic stroke when treated with either water or βHB solution.

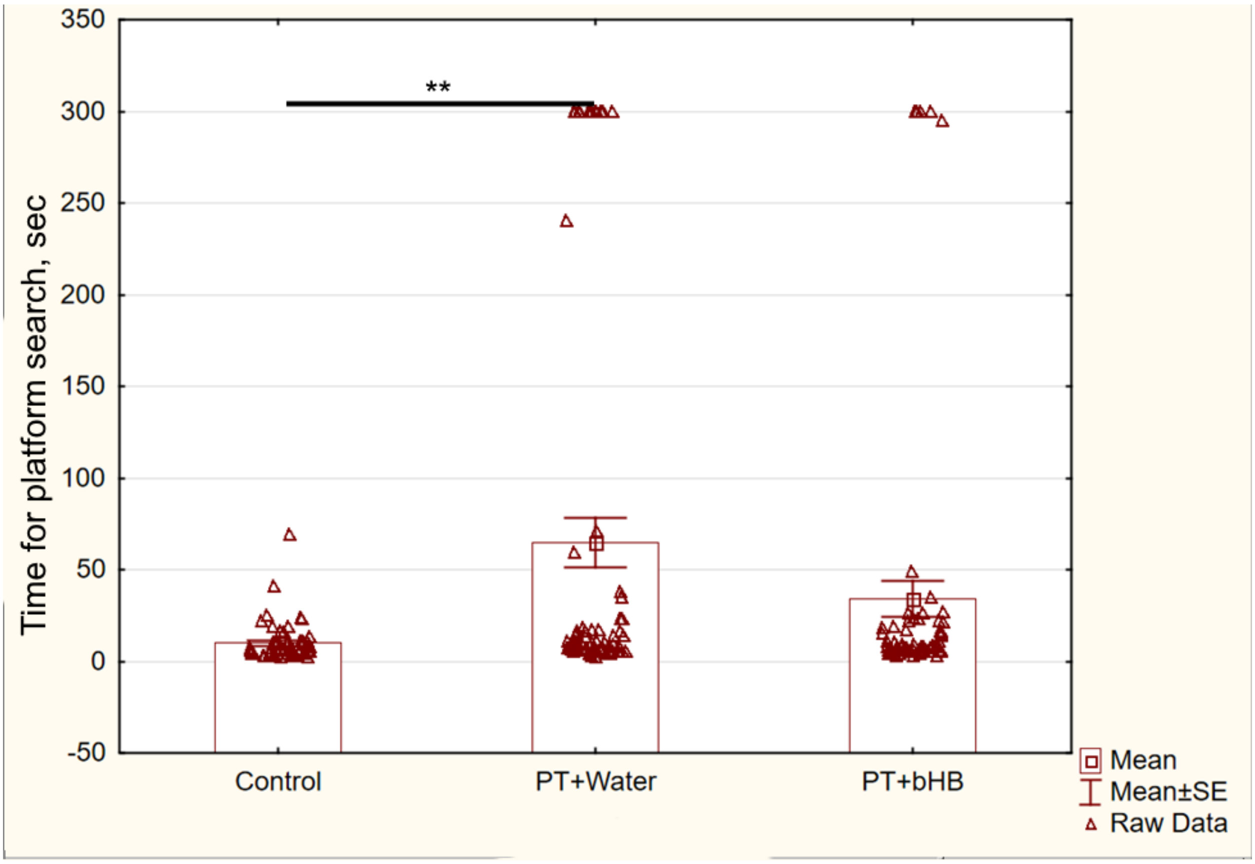

Supplementary Figure S2. Time spent by mice searching for the platform in the MWM test at the 2nd week after photothrombotic stroke when treated with either water or βHB solution.

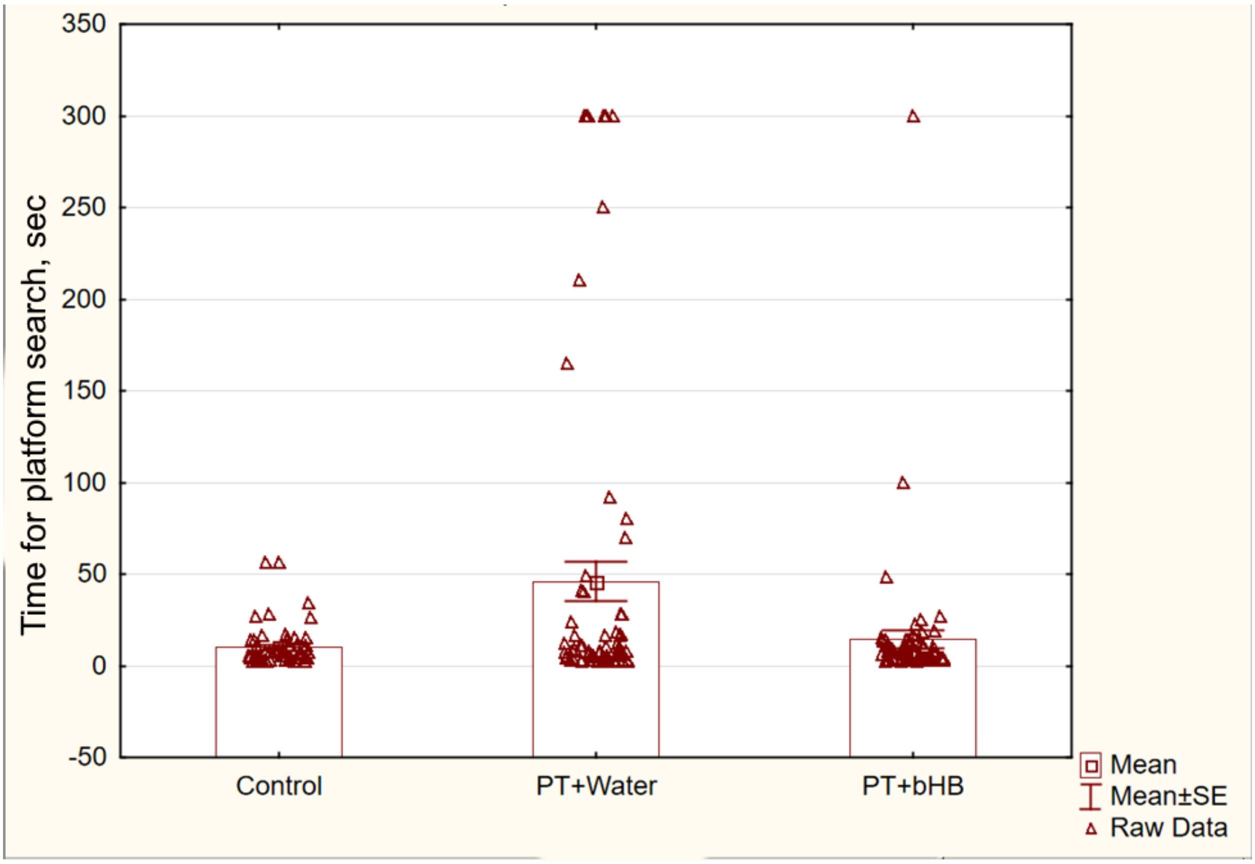

Supplementary Figure S3. Time spent by mice searching for the platform in the MWM test at the 3rd week after photothrombotic stroke when treated with either water or βHB solution.

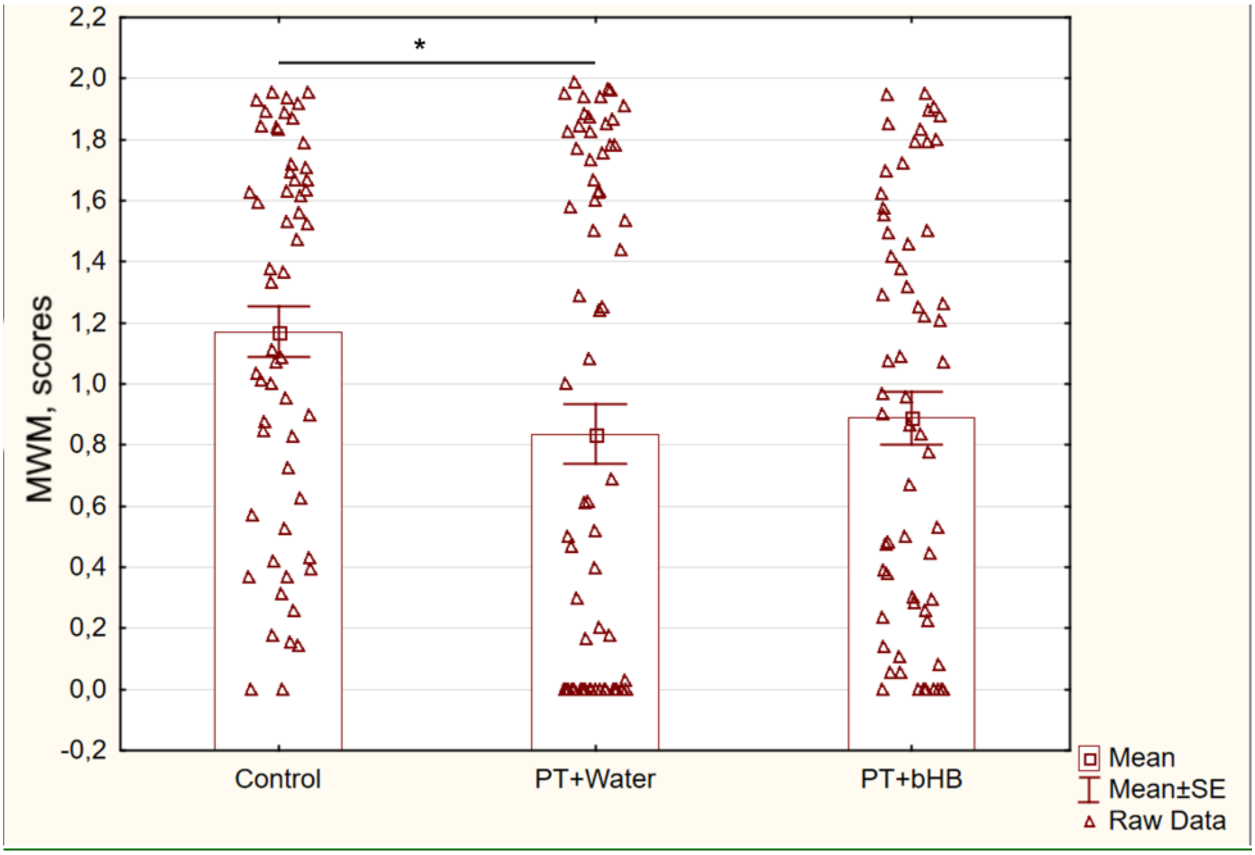

Supplementary Figure S4. Number of points scored by mice at the MWM test in the 1st week after photothrombotic stroke when treated with either water or βHB solution.

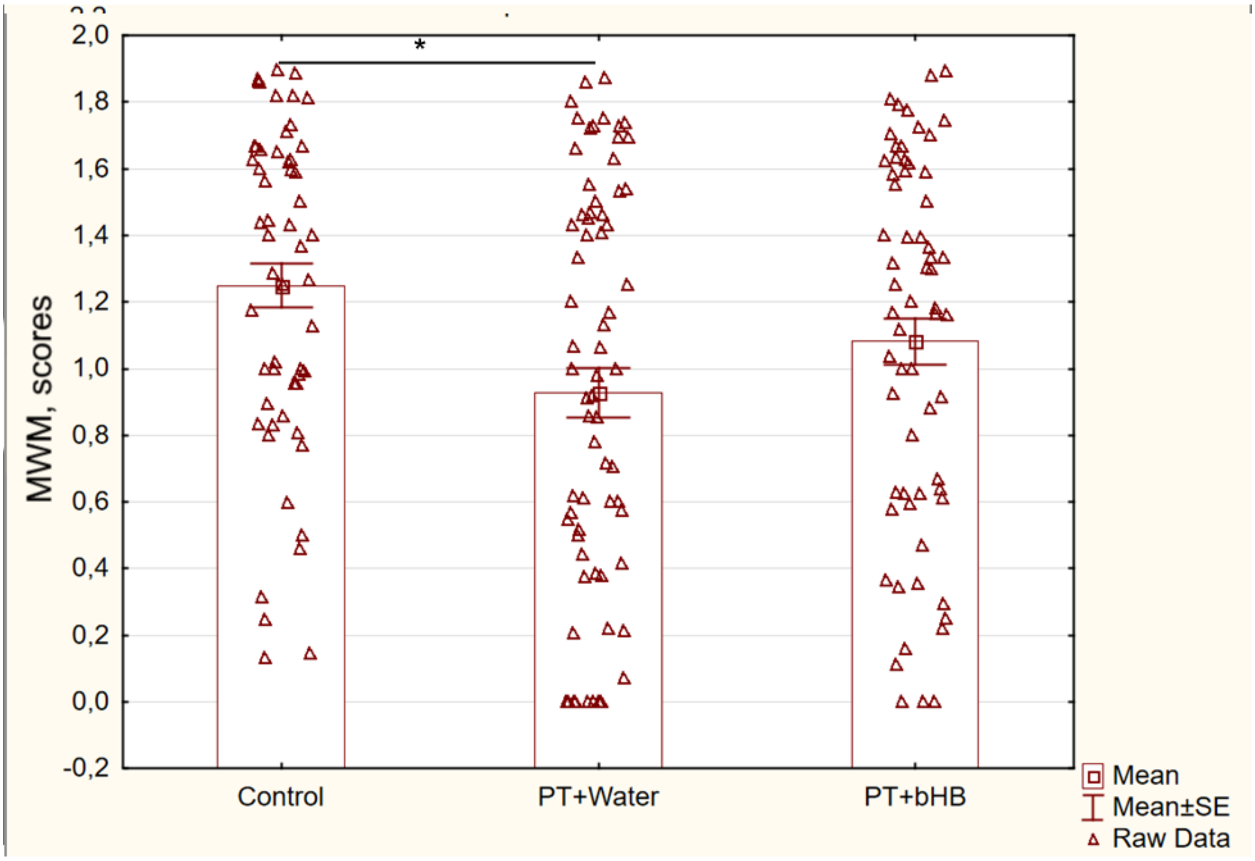

Supplementary Figure S5. Number of points scored by mice in the MWM test at the 2nd week after photothrombotic stroke when treated with either water or βHB solution.

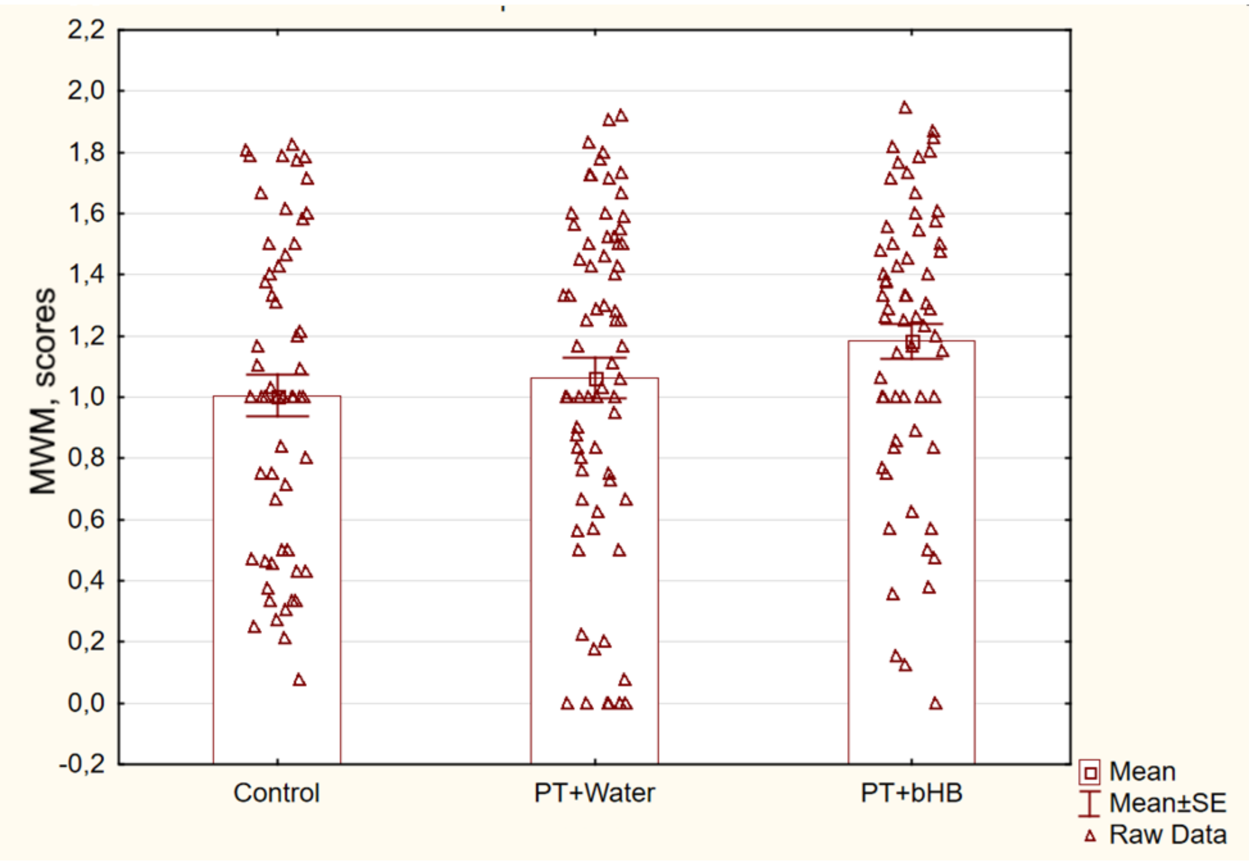

Supplementary Figure S6. Number of points scored by mice in the MWM test at the 3rd week after photothrombotic stroke when treated with either water or βHB solution.
